# MicroRNA expressing profiles in A53T mutant alpha-synuclein transgenic mice and Parkinsonian

**DOI:** 10.18632/oncotarget.13905

**Published:** 2016-12-11

**Authors:** Mingshu Mo, Yousheng Xiao, Shuxuan Huang, Luan Cen, Xiang Chen, Limin Zhang, Qin Luo, Shaomin Li, Xinling Yang, Xian Lin, Pingyi Xu

**Affiliations:** ^1^ Department of Neurology, The First Affiliated Hospital of Guangzhou Medical University, Guangdong, China; ^2^ Department of Neurology, The First Affiliated Hospital of Sun Yat-sen University, Guangdong, China; ^3^ Department of Neurology, The Third Affiliated Hospital of Xinjiang Medical University, Urumqi, China; ^4^ Ann Romney Center for Neurologic Disease, Brigham and Women's Hospital, Harvard Medical School, Boston, MA, USA; ^5^ Department of Anatomy & Guangdong Province Key Laboratory of Brain Function and Disease, Zhongshan School of Medicine, Sun Yat-sen University, Guangdong, China

**Keywords:** Parkinson's disease, A53T mutation, microRNAs, deep sequencing, Gerotarget

## Abstract

*α-synuclein* gene mutations can cause α-synuclein protein aggregation in the midbrain of Parkinson's disease (PD) patients. MicroRNAs (miRNAs) play a key role in the metabolism of α-synuclein but the mechanism involved in synucleinopathy remains unclear. In this study, we investigated the miRNA profiles in *A53T-α-synuclein* transgenic mice and analyzed the candidate miRNAs in the cerebrospinal fluid (CSF) of PD patients. The 12-month A53T-transgenic mouse displayed hyperactive movement and anxiolytic-like behaviors with α-synuclein aggregation in midbrain. A total of 317,759 total and 289,207 unique small RNA sequences in the midbrain of mice were identified by high-throughput deep sequencing. We found 644 miRNAs were significantly changed in the transgenic mice. Based on the conserved characteristic of miRNAs, we selected 11 candidates from the 40 remarkably expressed miRNAs and explored their expression in 44 CSF samples collected from PD patients. The results revealed that 11 microRNAs were differently expressed in CSF, emphatically as miR-144-5p, miR-200a-3p and miR-542-3p, which were dramatically up-regulated in both A53T-transgenic mice and PD patients, and had a helpful accuracy for the PD prediction. The ordered logistic regression analysis showed that the severity of PD has strong correlation with an up-expression of miR-144-5p, miR-200a-3p and miR-542-3p in CSF. Taken together, our data suggested that miRNAs in CSF, such as miR-144-5p, miR-200a-3p and miR-542-3p, may be useful to the PD diagnosis as potential biomarkers.

## INTRODUCTION

Parkinson's disease (PD) is characterized by dopaminergic neuronal degeneration with α-synuclein (SNCA) deposition in midbrain [1]. The α-synuclein (α-syn) physiologically regulates dopamine (DA) transmission by modulating synaptic transport and tyrosine hydroxylase activity [2]. Familial PD patients carried alanine-30→proline (A30P) or alanine-53→threonine mutation (A53T) of α-Syn have been well reported [3]. The transgenic mice expressing mutant A53T α-Syn were constructed for PD study [4–12]. These transgenic model with over-expression of wild type or A53T α-Syn showed PD-like symptoms and synaptic impairment [4, 9, 10, 13–20]. For example, the hemizygous Prnp-SNCA*A53T mice mixed with C57BL/6J×C3H, were found the elevated levels of α-Syn and phosphorylated-tau in striatum and 30.7% DA neurons decreased in Substantia nigra (SN) [5]. Their homozygous offspring, backcrossed to a C57BL/6J background, had age-dependent abnormalities such as hyperactive movement, anxiolytic-like behaviors, motor and sensorimotor deficits [4]. These non-motor and sensorimotor deficits were prior to the gross motor dysfunction [1, 4, 13]. It suggested that A53T mutant model share similar pathogenesis mechanism with clinic PD.

Non-coding RNA (ncRNA) was found to be involved in α-synuclein pathogenesis [21]. MicroRNAs (miRNAs), or short ncRNAs, regulate the translation or degradation of target messenger RNAs (mRNAs) at the post-transcriptional stage [22]. For example, miR-7, -433 and -153 bind to the 3′-untranslated region of *α-synuclein* to inhibit the gene expression in DA neurons [23, 24]. The mutant α-synuclein is more difficult to be degraded than wild-type by the ubiquitin-proteasome system [25]. Thus, it should be more important to explore the miRNA profiles in mutant α-synuclein than wild-type to evaluate protein aggregation in PD [26]. Recent reports found that some miRNAs can be packaged into lipid-based carriers and stable in the plasma, cerebrospinal fluid (CSF) and urine [27, 28]. Down regulation of miR-16-2-3p and -1294, up regulation of miR-338-3p, -30e-3p, and -30a-3p were found in the plasma or CSF of PD patients [29, 30]. These miRNAs may be novel biomarkers for PD diagnosis and prognosis. However, the miRNA signatures of PD remain unclear to date. In this study, we attempted to screen the miRNAs profiles in A53T-transgenic mice and evaluate their value for the clinical diagnosis of PD.

## RESULTS

### A53T-transgenic mice display hyperactive behavior with increased α-synuclein deposition in the degenerating DA neurons

**Figure 1:**
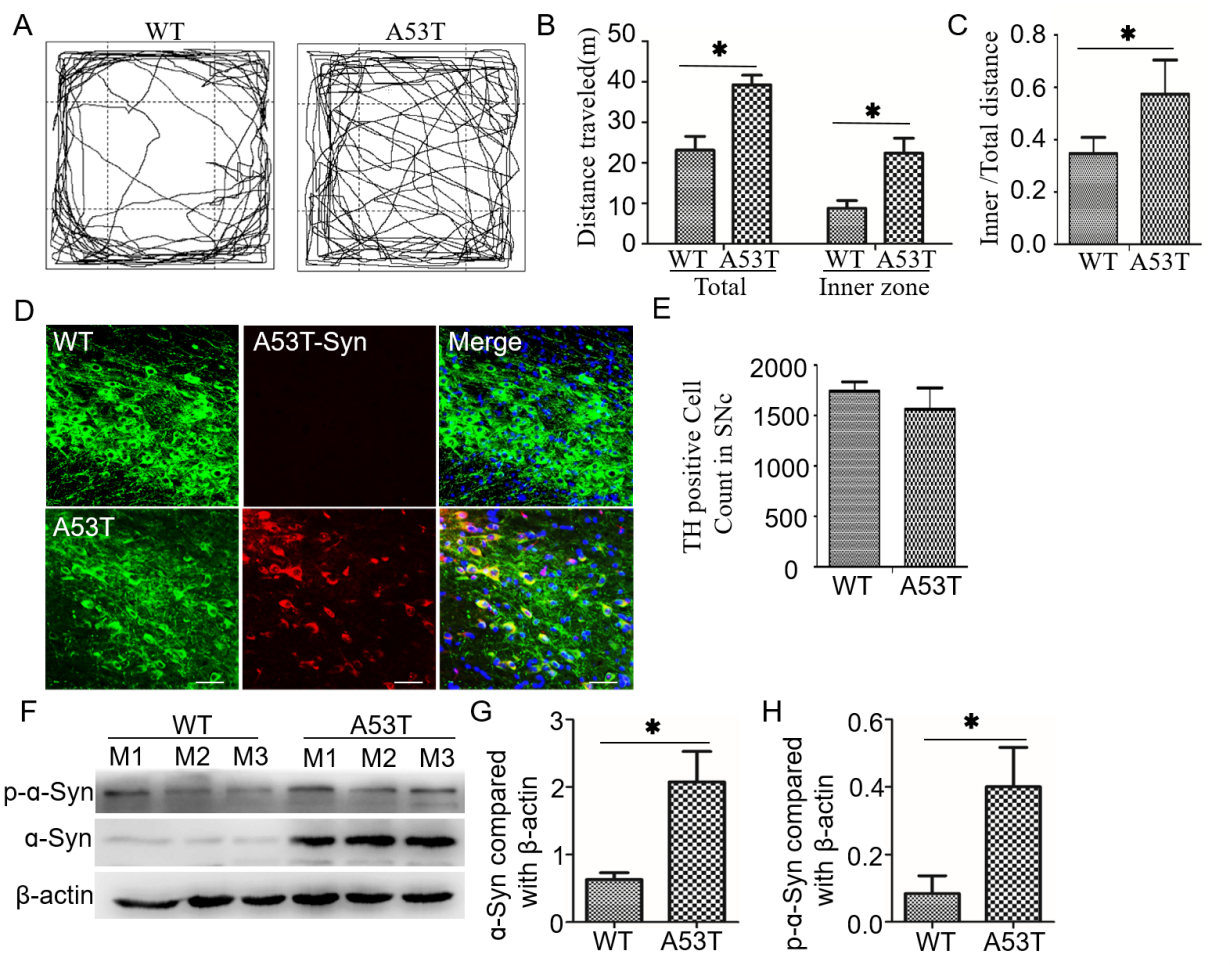
A53T mice show increased movement, decreased dopaminenergic neurons and increased α-synuclein aggregation in the midbrain **A.** In an open-field test, A53T-α-synuclein mice displayed hyperactive movement at 12 months of age. **B.** The distances traveled in the total field and inner field in 20 min were compared between A53T-transgenic and wild-type mice (*n* = 6). **C.** The ratio of inner field to the total field was increased in A53T mice compared with wild-type mice. **D.** A53T-α-synucleins in the midbrain (arrows) were labeled with red fluorescence under immunofluorescence double-staining, and the TH-positive neurons were stained with green fluorescence. **E.** The number of TH positive neurons is accounted in SN. **F.** Levels of α-synuclein and p-α-synuclein were detected in midbrains by western blot analysis. The three mice in each group were labeled as M1 to M3. Histograms showing the difference in total α-synuclein **G.** and p-α-synuclein **H.**. All data are expressed as the mean ± SD, *P < 0.05, the Wilcoxon-Mann-Whitney test was used for the behavior test and the Student t test for the rest comparison.

The behavior of mice at 12 months of age was tested using the open field test. The A53T-transgenic mice displayed hyperactivity, as indicated by a longer distance traveled in the center region (Figure 1A). The distances moved within 25 min by A53T mice and wild-type mice were 3,721.73 ± 238.81 cm and 2,181.74 ± 290.50 cm (*p* < 0.05) respectively. The inner distances moved by A53T mice and wild-type mice were 2,138.37 ± 365.92 cm and 975.01 ± 184.93 cm (*p* < 0.05, Figure 1B) respectively. Thus, the ratio of inner/total distances in A53T was significantly higher than that of in wild-type mice (0.58 ± 0.17 *vs*. 0.34 ± 0.08, *p* < 0.01, Figure 1C). It suggested that A53T-transgenic mice display anxiolytic-like and hyperactive behaviors. The immunofluorescence analysis indicated that a dense distribution of mutant α-synuclein particles was commonly observed at the DA neurons of A53T-transgenic mice but rarely found in wild-type (Figure 1D). The quantitative cell analyses revealed a slight decrease without significance in DA count in the SN of A53T mice (1741 ± 94.18 cells in wild-type *vs*. 1560 ± 213.72 cells in A53T, *p* = 0.064, Figure 1E). Western blot analysis of midbrain revealed the total and phosphorylated α-synuclein increased significantly in the mutant mice compared to the wild-type (*p* < 0.001, Figure 1F-1H).

**Table 1:** Clinical data of PD patients and healthy control

Variable		Control (n=42)		PD (n=44)		p-value (PD vs. control)
		NO.	%	NO.	%	
Average age (mean ± SD, years)		55.42 ± 12.50		56.83 ± 17.12		0.67
Age(years)	≥55	23	54.76	27	61.36	0.54
	<55	19	45.24	17	38.64	-
Gender	Male	24	57.14	26	59.09	0.85
Smoking	Current	7	16.67	5	11.36	0.40
	Ever	19	45.24	19	43.18	-
	Never	16	38.09	20	45.45	-
Age at onset (years)		-		56.42 ± 7.14		-
Disease duration(years)		-		2.23 ± 1.27		-
H&Y scale	1.0	-		12	27.27	-
	1.5	-		8	18.18	-
	2.0	-		14	31.82	-
	2.5	-		10	22.73	-
UPDRS part-III		-		18.50 ± 1.53		-

### miRNA signature in A53T-transgenic mice

**Figure 2:**
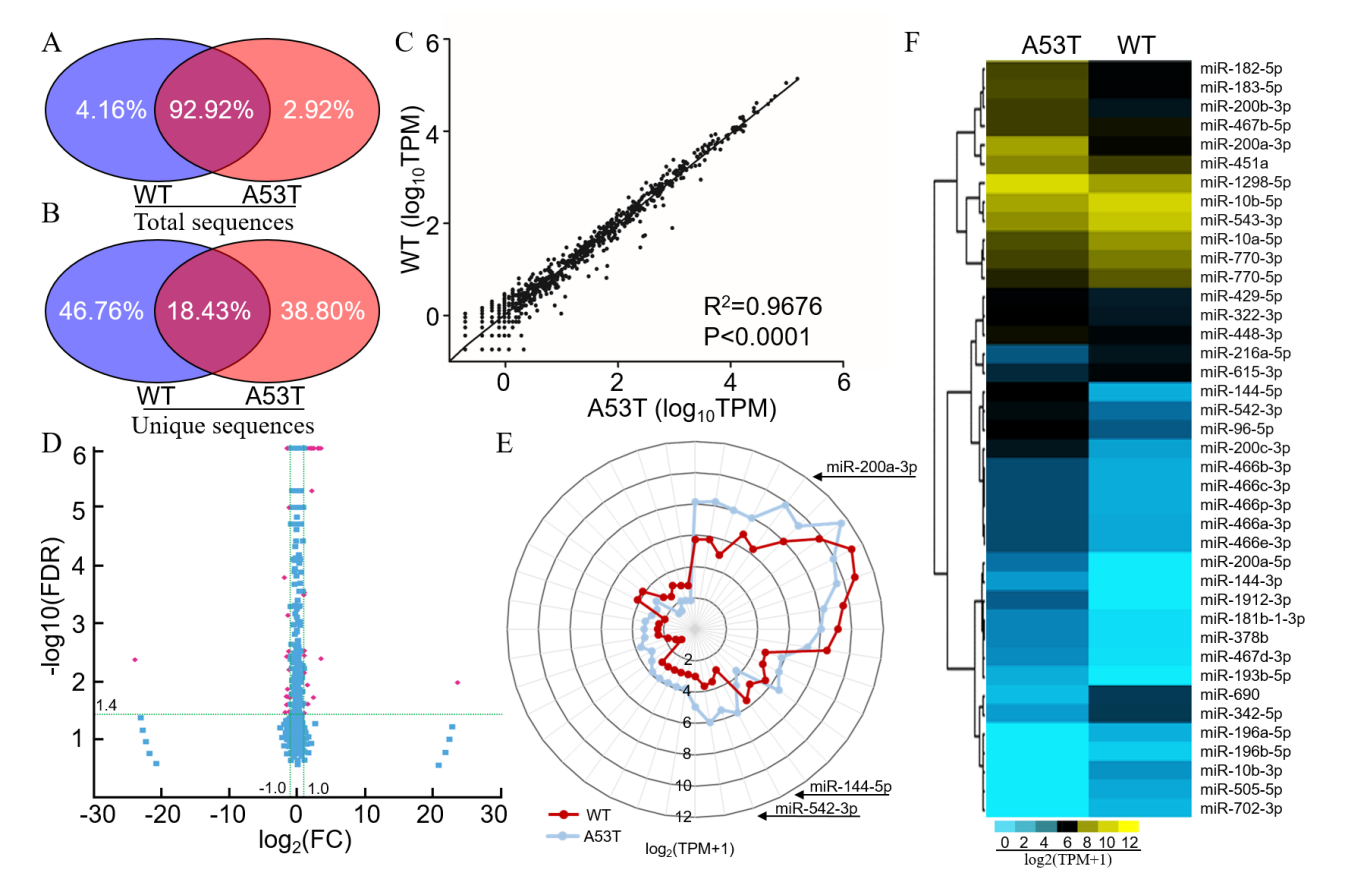
A53T-transgenic mice exhibited a distinct miRNA signature in the midbrain Distribution of total miRNA sequences **A.** and specific sequences **B.** of the midbrain in A53T-transgenic (left, blue) and wild-type mice (right, red). **C.** Correlation of miRNAs based on a Spearman non-parametric analysis. TPM (transcripts per million) = (Readcount × 1,000,000) / Mapped Reads. **D.** Volcano plot showing the different miRNAs in A53T-transgenic mice compared to wild-type mice. Red dots indicate a fold-change expression > 2 (|log2 FC| > 1) and *P* < 0.05 [−log10 (FDR) > 1.4]. FC: Fold Change, FDR: False Discovery Rate. **E.** Star glyph of differentially expressed miRNAs in A53T-transgenic and wild-type mice. The miRNAs contain 40 differentially expressed miRNAs form the results of volcano plot. The axis ratio followed the log_2_ (TPM+1) scale. The red and blue curve represented the A53T and wild-type groups. The miRNAs order in Star glyph was same as in hierarchical clustering heatmap. TPM: Transcripts per million clean tags. **F.** Hierarchical clustering heatmap of miRNAs from star glyph showing comparisons between A53T-transgenic and wild-type mice. The color range gradient from green to red represents the abundance of miRNAs.

Small RNA (sRNA) libraries from the midbrain of A53T-transgenic mice were analyzed using the Illumina HiSeq2500 platform. A total of 12,334,900 and 10,916,235 clean reads were monitored in the A53T-transgenic and wild-type mice, respectively. We excluded the sequences of null or poor-quality 3′ insert nucleotides with sequence lengths > 30 or < 18 nt. The majority of sRNAs were 21-24 nt, which contained the miRNAs (Supplementary Figure. 2). These RNAs were divided into several sub-categories, including rRNAs, scRNAs, snRNAs, snoRNAs, tRNAs, repbase, and unannotated RNAs (Supplementary Table 2). By high-throughput sequencing, 317,759 (2.92%) of the total sRNA sequences or 289,207 (34.80%) of the unique sRNA sequences were uniquely detected in the A53T-trangenic mice, 452,631 (4.16%) of the total sRNA sequences and 388,626 (46.76%) of the unique sRNA sequences were detected in wild-type mice. There was an overlap in 10,107,585 (92.02%) of the total sequences and 153,195 (18.43%) of the unique sequences (Figure 2A and 2B). Six hundred and forty four unique miRNAs were significantly different between A53T-transgenic and wild-type mice (R^2^ = 0.97, *p* < 0.0001, Figure 2C). Among them, 32 miRNAs were up-regulated and 25 miRNAs were down-regulated with a fold change ≥ 2 (*p* < 0.05, Figure 2D). In this sequencing, miR-9-5p and miR-7a-5p were most abundantly expressed in the A53T mice [Transcripts per kilobase million (TPM) = 153248.90 and 97029.90, respectively], but the expression was not changed dramatically compared with WT mice (fold-changes of 0.90 and 1.15, respectively). The miR-200a-3p and miR-1306-3p had the most significantly changed fold (fold-changes of 11.62 and 11.51, respectively), but the miR-1306-3p has a low expression in A53T and WT mice (TPM = 2.10 and 0.18, respectively). Excluded the miRNAs with low expression level such as miR-1306-3p and miR-7790-3p, 40 miRNAs are displayed as a special signature in a star glyph distribution (Figure 2E), and each distinct miRNA profile is shown in a hierarchical clustering heatmap (Figure 2F).

### Target genes of A53T-related miRNAs

The 641 known and 3 novel miRNAs in sequencing result were used to predict the targeted genes. The 22,160 genes targeted by miRNAs were analyzed using the miRanda and RNAhybrid prediction databases. To annotate these target genes, the clean reads of miRNAs were searched using the BLAST database. A total of 20,116 target genes were annotated. Following the 2-fold-change cut-off (*p* < 0.01), 5,395 genes were subjected to the COG database, 18,293 genes were subjected to the partial GO database, 9,783 genes were subjected to the KEGG database, 19,393 genes were subjected to the Swissprot database and 20,116 genes were subjected to non-redundant (NR) databases.

GO enrichment analysis categorized the miRNA target genes into three enriched fields: (i) binding, catalytic activity and molecular transducer activity in molecular function field; (ii) cellular processes, biological regulation and metabolic processes in the biological process field; (iii) cell part, cell and organelle in the cellular component field. The subdivided functional annotation analysis by TopGO revealed that the most enriched GO terms were involved in the positive regulation of apoptotic processes [GO: 0043065, KS (Kolmogorov-Smirnov test) = 5.10 × 10^−17^], ATP binding (GO: 0005524, KS < 1 × 10^−30^) and protein binding processes (GO: 0005515, KS < 1.0 × 10^−30^). Other interestingly enriched GO terms included aging (GO: 0007568, KS = 8.20 × 10^−10^) and protein metabolism as autophosphorylation (GO: 0046777), heterooligomerization (GO: 0051291, GO: 0042803), identical protein binding (GO: 0042802), domain specific binding (GO: 0019904), kinase binding (GO: 0019901), complex binding (GO: 0032403), and ubiquitin protein ligase (GO: 0031625). These enriched miRNAs were closely associated with the mitochondria (GO: 0005739, KS = 1.80 × 10^−23^) and cytoplasm (GO: 0005737, KS = 1.01 × 10^−30^, Table 2). The major putative COG-annotated proteins were predicted for replication, recombination and repair, transcription, and signal transduction. The biological interpretation by KEGG analysis revealed that the most frequent pathways included neuroactive ligand-receptor interactions [ko04080, EF (Enrichment factors) = 0.95, *p* < 0.001], focal adhesion (ko04510, EF = 0.94, *p* < 0.001), gap junction (ko04540, EF = 0.93, *p* < 0.001), protein digestion and absorption (ko04974, EF = 0.93, *p* < 0.001), and axon guidance (ko04360, EF = 0.93, *p* < 0.001, Supplementary Table 3). Among them, 99 target genes were associated with PD, according to the annotated information in the KEGG analysis (Supplementary Figure 3).

**Table 2:** Targets of miRNAs involved in the A53T mutant protein predicted by GO enrichment analysis

Classification	Pathway Name	GO.ID	Genes Regulated by miRNAs			-In (*P* value)
			Annotated	Significant	Expected	
Molecular function	ATP binding	GO:0005524	1635	1573	1509.70	30
	Protein binding	GO:0005515	8556	7991	7900.28	30
	Zinc ion binding	GO:0008270	1219	1159	1125.58	17
	Protein homodimerization activity	GO:0042803	880	827	812.56	14
	Protein domain specific binding	GO:0019904	850	802	784.86	13
	Identical protein binding	GO:0042802	1336	1262	1233.61	12
	Protein serine/threonine kinase activity	GO:0004674	496	482	457.99	11
	Protein kinase binding	GO:0019901	579	552	534.63	10
	Sequence-specific DNA binding transcript	GO:0003700	974	933	899.35	9
	Ubiquitin protein ligase binding	GO:0031625	191	178	176.36	9
Biological process	Positive regulation of apoptotic process	GO:0043065	473	446	437.63	17
	Response to drug	GO:0042493	769	725	711.49	15
	Positive regulation of transcription factor activity	GO:0045944	993	950	918.74	15
	Response to hypoxia	GO:0001666	424	403	392.29	11
	Protein autophosphorylation	GO:0046777	248	242	229.45	11
	Positive regulation of transcription	GO:0045893	1353	1285	1251.82	10
	Positive regulation of cell proliferation	GO:0008284	948	884	877.11	10
	Protein transport	GO:0015031	1443	1339	1335.09	10
	Aging	GO:0007568	412	387	381.19	10
	Protein heterooligomerization	GO:0051291	139	130	128.61	9
Cellular component	Cytoplasm	GO:0005737	10368	9614	9540.98	30
	Mitochondrion	GO:0005739	2040	1864	1877.28	23
	Nucleolus	GO:0005730	1719	1597	1581.88	20
	Nucleus	GO:0005634	6630	6131	6101.15	19
	Cytosol	GO:0005829	1715	1557	1578.20	18
	Golgi apparatus	GO:0005794	1340	1255	1233.11	16
	Neuronal cell body	GO:0043025	628	588	577.91	16
	Perinuclear region of cytoplasm	GO:0048471	674	636	620.24	14
	Lamellipodium	GO:0030027	159	155	146.32	11
	Membrane raft	GO:0045121	357	343	328.52	11

The numbers of genes annotated to the term in the background and input data are shown in the “Annotated” and “Significant” columns, respectively. The number in the “Expected” column represents the genes expected in the input data by chance. The *p*-value was used to evaluate the term enrichment after adjusting the false discovery rate.

### Candidate biomarker of miRNAs in PD patients

**Figure 3:**
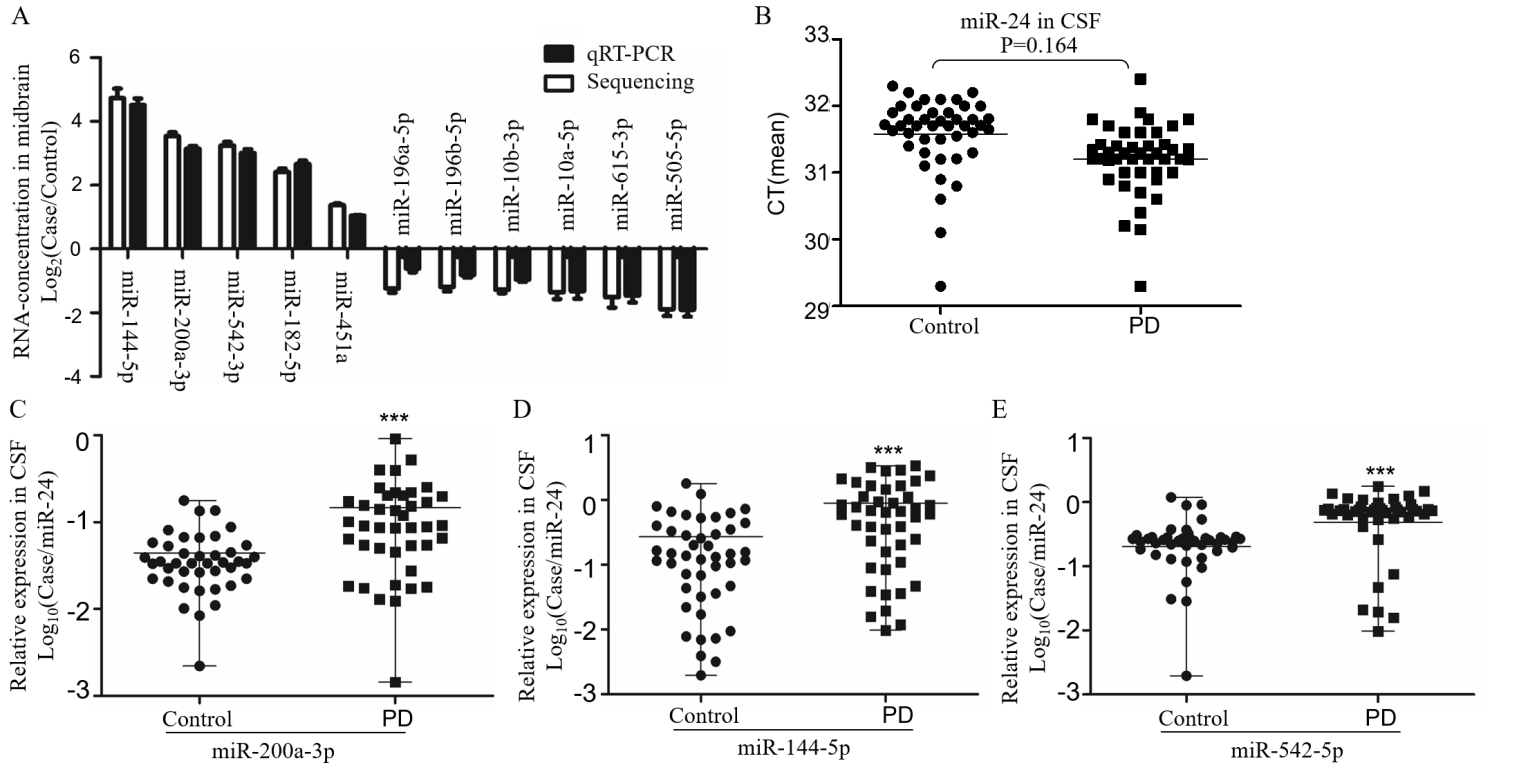
The expression of candidate miRNAs in CSF from PD **A.** The sequencing result of miRNA concentration confirmed by qRT-PCR in the midbrain of A53T-transgenic mice (*n* = 5). The miRNAs with significant different expression were shown in the histogram, data were expressed as mean ± SE, with corrected *P* < 0.05 in Wilcoxon-Mann-Whitney test. **B.** The CT value of miR-24 in CSF was used to evaluate its possibility as control. The relative expression of miR-200a-3p **C.**, miR-144-5p **D.** and miR-542-3p **E.** in CSF from PD and health controls by qRT-PCR. Data expressed as mean ± range. ***P < 0.001 after Bonferroni correction for 11 tests.

**Figure 4:**
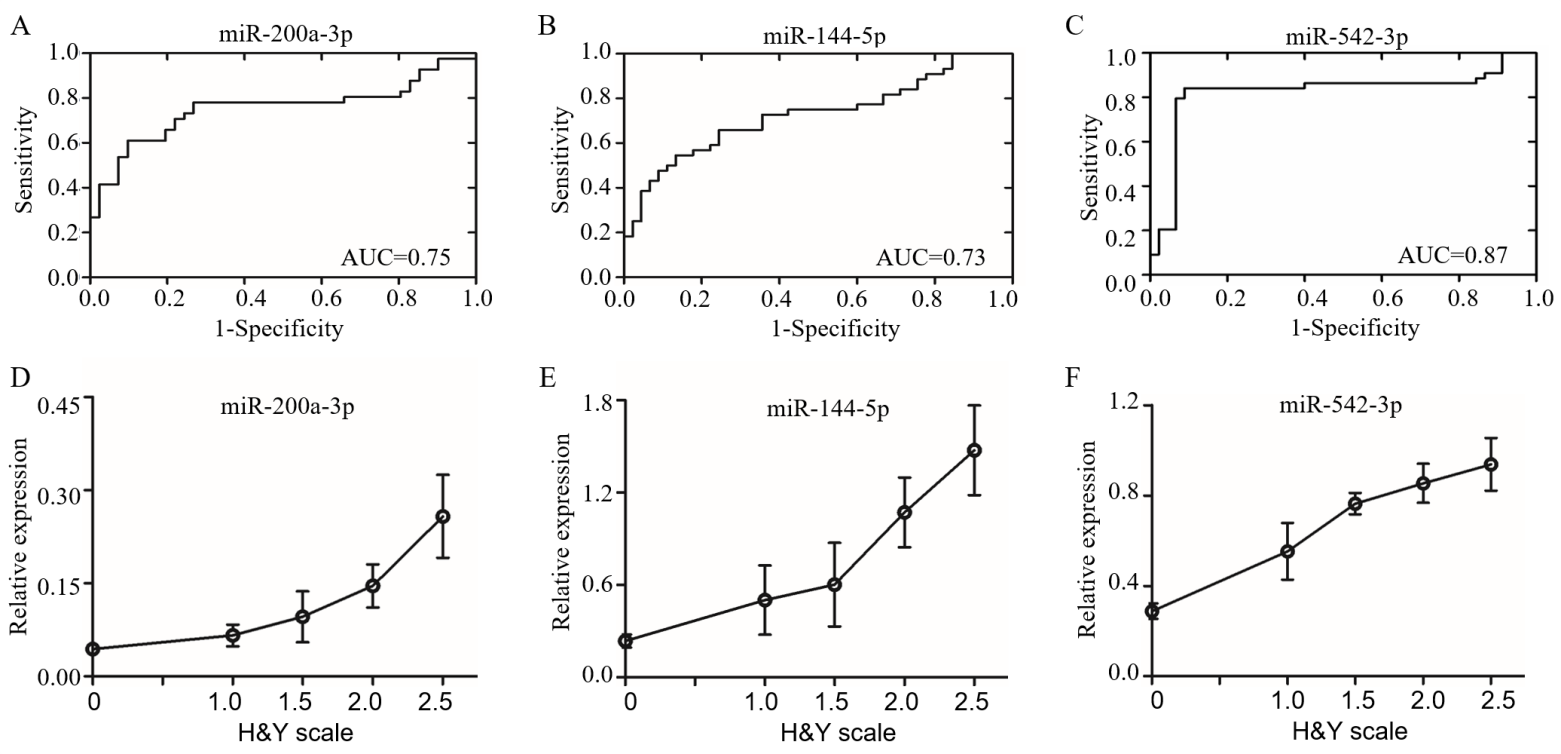
The miRNAs in CSF as candidate biomarkers of PD The feasibility of miR-200a-3p **A.**, miR-144-5p **B.** and miR-542-3p **C.** in CSF for PD diagnosis were assessed by receiver operator characteristics (ROC) analysis. The miR-200a-3p **D.**, miR-144-5p **E.** and miR-542-3p **F.** were increased in CSF form PD and changed with H&Y scale. The Y axis was the relative expression for each miRNA, while the X axis represented H&Y scale. Data expressed as mean ± SE. AUC: area under ROC curve, H&Y scale: Hoehn and Yahr scale.

Recent studies showed that miRNAs are stable in human body fluid [31, 32]. Based on the conserved miRNA sequences (Supplementary Table 4), we screened the candidate miRNAs in the CSF samples from PD patients with same primers by qRT-PCR. The levels of candidate miRNAs were first confirmed in A53T mice (Figure 3A). We recruited 44 PD patients at early stage without levodopa treatment and with age and gender matched 42 health controls (Table 1). The reported stable miR-24 was selected as the internal control (Figure 3B) [33]. The miR-196a-5p, -196b-5p, -10b-3p, -10a-5p, -615-3p, and -505-5p were down-regulated, whereas miR-144-5p, -542-3p, -200a-3p, -182-5p and -451a were up-regulated. The most significant up-regulating miRNAs were miR-144-5p, -200a-3p and -542-3p, as their fold-changes were 3.15 ± 0.08, 3.01 ± 0.12 and 2.66 ± 0.11 respectively in the mouse brain, and 3.24± 0.50, 3.63 ± 0.57 and 2.66 ± 0.19 respectively in the CSF of PD patients (Figure 3C-3E). The miR-144-5p, miR-200a-3p and miR-542-3p were evaluated for the PD diagnose. Their receiver-operating characteristic (ROC) curve areas were 0.73 (95% CI: 0.62-0.84), 0.75 (95% CI: 0.64-0.87), and 0.87 (95% CI: 0.72-0.92) respectively. At the cut-off values of 0.35 for miR-144-5p, 0.05 for miR-200a-3p, and 0.40 for miR-542-3p, the sensitivity and specificity for these markers were 65.91% and 75.56%, 73.17% and 75.61%, 84.09% and 91.11%, respectively (Figure 4A-4C). The database was classified as 5 groups by Hoehn and Yahr (H&Y) scales when healthy control were classified as 0 scale. The expression of miR-144-5p, miR-200a-3p and miR-542-5p showed an increased tendency accompanied with high H&Y scales of PD patients (Figure 4D-4F). The ordinal regression analysis was used to investigate the correlations. The age, gender, smoking and H&Y scales were included as independent variables. As a result, the candidate miRNAs were significant increased following the PD severity with the coefficient: 12.51 (95% CI: 7.51-17.51) in miR-200a-3p, 1.33 (95% CI: 0.74-1.92) in miR-144-5p, 4.64 (95% CI: 3.05-6.52) in miR-542-3p respectively (Table 3). Correspondingly, the gender, age and smoking did not significantly contribute to the expression difference of miR-200a-3p (*p* = 0.69, 0.34 and 0.03), miR-144-5p (*p* = 0.47, 0.19 and 0.83) and miR-542-3p (*p* = 0.56, 0.65 and 0.65, Supplementary Figure 4).

**Table 3:** The CSF miRNAs showed progressive expression trends across increasing H&Y scales by ordinal regression analysis

MiRNAs	Coefficient	SE^*^	P>(z)	H&Y thresholds (95%CI)			
				H&Y:1	H&Y:1.5	H&Y:2	H&Y:2.5
miR-200a-3p	12.51	2.55	<0.001	0.34-1.56	1.05-2.45	2.05-3.91	2.84-5.13
miR-144-5p	1.33	0.30	<0.001	0.28-1.45	0.88-2.19	1.78-3.45	2.48-4.61
miR-542-3p	4.64	0.81	<0.001	1.34-3. 20	2.13-4.34	3.08-5.63	3.89-6.76

* SE: the standard error of coefficient.

## DISCUSSION

In this study, we used A53T-transgenic mice as PD model to describe the miRNA signatures of midbrain in PD and predict the miRNAs functions. In the miRNA signatures of PD, we found the miRNAs with dramatic change of expression, included miR-196a-5p, -196b-5p, -10b-3p, -10a-5p, -615-3p, -505-5p, -144-5p, -542-3p, -200a-3p, -182-5p and -451a that expressed in the body fluids and associated with mutant α-synuclein aggregation. Among them, we confirmed that miR-200a-3p, -144-5p and -542-3p had significantly higher concentration in the CSF from PD patients. The ROC analyses and ordinal logistic regression model further supported the miR-200a-3p, -144-5p and -542-3p might be served as biomarkers for PD diagnosis at the early stage.

The A53T mutation of *α-synuclein* gene was reported in Greek familial PD patients [34]. Then a series of A53T transgenetic models were developed for PD study. These models showed different symptoms and pathologies with different promoters, genotypes and mouse lines [4, 9, 16, 35–37]. The A53T transgenic mice, applied Thy-1 promoter, were found the amorphous aggregates of human α-synuclein accompanied with paralysis-like motor dysfunction, but no DA neurons lost in SNc [38, 39]. Another transgenic B6 mice, applied the prion protein (Prp) promoter, were used to promote *α-synuclein* expression and show an increased neurotoxicity in nervous system [40, 41]. As to the genotype factor, the homozygous offspring of A53T mice develop some paralysis-like symptoms and die approximately at 16-month old as reported by Jackson Laboratories, but the heterozygous offspring rarely show the similar pathology signs and the onset time will has 22-28 months delay [9, 42, 43]. To mimic the impaired SN system on PD, other Prp-A53T model, used C3H mice mixed with C57BL line, was constructed. These transgenic mice showed that TH-positive cells decreased significantly in SN accompanied with α-synuclein deposition without the paralysis-like symptoms [4, 5]. Here, we used the hemizygous Prp-A53T offspring derived in the C57BL/B6 background and confirmed these transgenetic mice developed some abnormalities included the mutant-α-synuclein aggregation, increased *p*-α-synuclein and hyperactive movements. Their behavior abnormalities, such as hyperactive movements and anxiolytic, were token as non-motor symptoms in the preclinical stage of PD [4, 18, 39]. Our mice had the similar background as Paumier's and Wills' study, but their DA neurons in SN are not dramatically decreased as report [4, 5]. It may be related with the preclinical or early stage of disease and young age on 12-month-old A53T mice.

The non-motor symptoms, such as dysautonomia, sensory dysfunctions and behavioral abnormalities, appear earlier than motor symptoms at the early stage of PD [44, 45]. The symptoms like anxiety and depressive disorders at the preclinical phase are taken as risk factors for PD [46]. The anxiety and depressive behaviors are commonly evaluated by open field test in animal models [47]. In our study, the open filed test was also used and the result showed the heterozygous A53T mice took more time and higher ratio in the center region with hyperactive motor, which was also found in the homozygous mice on Katrina L's study [4]. These data supported that A53T mice may develop an anxiolytic phenotype [4, 16, 18, 39, 41, 48]. Although anxiolytic is not like the signs and symptoms in PD, it should be emphasized that A53T mutant is only one of PD-related factors and anxiety is a complex disorder involved with multiple factors [49]. A study suggested this hyperactivity acts as an abrupt appearance, but not a progressive impairment in motor ability [4]. It may be involved with the increased sensitivity of DA neurons in midbrain, which is caused by a series of functional disorders included the decreased DA transporter expression, impaired striatal DA uptake and elevated D1 receptor expression [16, 36, 37, 50]. In a word, the anxiolytic-like phenotype reflect the abnormality of dopaminergic nigrostriatal system after A53T mutant. Other non-motor symptoms, such as olfactory dysfunction, were also found in the A53T mice 6 months before the motor deficits and DA neurons impairment [10, 14, 41]. These evidences suggested that A53T model may share some similar symptoms and mechanism of the preclinical stage of PD. And the A53T mice may provide meaningful diagnostic markers at early stage before the motor symptoms.

The mechanism of miRNAs involved in α-synuclein aggregation is still unclear. A few miRNAs were reported to be interacted with PD-related genes in PD pathogenesis. For example, miR-7 regulates the expression of A53T *α-synuclein* [51], miR-7 and -153 participate in SNCA transcription [23, 51], the function of miR-1224, -184 and let-7i-3p/5p is regulated by leucine-rich repeat kinase 2 (LRRK2) [52, 53], and miR-127-5p and -16-5p play their roles in glucocerebrosidase (GBA) pathway [54]. Several miRNAs were found with different expression levels in PD models, such as miR-135a-5p, -214 and -124 in 1-methyl-4-phenyl-1, 2, 3, 6-tetrahydropyridine (MPTP) mice [55–58], miR-155 in AAV-α-Syn PD mice [59], and miR-10a, -10b, -212, -132, -495 in A30P tg mice [26]. In PD patients, the deficient expressions was demonstrated on miR-141, -214, -146b-5p, -193a-3p, -133b, -433, -34b/c and -205 [24, 60–63], and other different signatures of 29 miRNAs in brain [64]. Here we used deep sequencing technique for miRNAs analysis, this technical method is independent of the miRNA database and provides better accuracy and precision on miRNAs detection [65]. Our data revealed that more than 644 miRNAs were detected with significant level-change in A53T mice, especially 40 miRNAs with fold-change ≥ 2. Among the 40 miRNAs, miR-542-3p, -10a-5p, -10b-3p, -141, -200b-3p, -542-3p, and -505-5p were confirmed with similar fluctuation of expression as reports [26, 30, 66–68]. More importantly the miR-200a-3p and -144-5p were showed the different expression in PD mice for the first time. Other miRNAs reported by several PD studies, such as miR-133b, -34c-5p, -205,-155, -135b, -212, -495, -193a-3p, -34b/c and -485-5p, shared the similar expression regulation with our result, but their fold-change was lower and didn't meet our inclusion criteria [59, 61–63, 68–70]. However, the miR-214, -124, -132, -127-5p, -146b-5p, and -16-5p reported in other PD models, were not found the different expression [26, 30, 54, 55, 57, 60]. And some PD-related miRNAs, included miR-7, -153, -1224, -184 and let-7i-3p, were just changed slightly in our results [23, 51–53]. In a word, our sequencing result indicated a series of miRNAs can be potentially considered as new biomarkers for PD prediction although most reported miRNAs have been confirmed in our investigation.

The bioinformatics analyses further indicated that the miRNA signatures in A53T mice may contribute to the predicted functional network, including the positive regulation of apoptotic processes, ATP binding biological processes, protein binding, protein-related functions and metabolism, neuroactive ligand-receptor interactions and aging. Among them, 99 target genes were found to be related to PD. Due to miRNA binding sites are short, a little difference in the algorithms will render the dramatic diversity, the bioinformatics prediction of miRNA targets is hard to avoid some disadvantages, such as false positives and false negatives, or inconsistent results using different algorithms, and the predicted targets might not be expressed in specific conditions [65, 71]. To avoid these drawbacks, we integrated the prediction by a combination with our founding and other experimental results and reports. The bioinformatics result first was partly supported by the pathology of α-synuclein inclusions and decreased DA neurons in PD (Figure 1). The α-synuclein inclusions impaired the ubiquitin-proteasome system as major protein degradation pathways [72], aggregated at endoplasmic reticulum (ER) and induced ER stress [73], accompanied with ATP depletion and oxidative stress related with neuron apoptosis [74, 75]. These PD-related molecular mechanisms were mostly overlap with the predicted functional of miRNA signature in A53T mice.

Several miRNAs, embedded in lipid or as lipoprotein complexes, were reported to be detectable and stable in CSF and serum [31]. Seventy-three percent of the distinct miRNAs in Alzheimer's disease (AD) brain can be detected in CSF [29]. Seventeen miRNAs in the CSF and five miRNAs in the serum were found in PD patients at abnormal level [30], suggesting that miRNAs in CSF or serum may be potential biomarkers of neurodegenerative diseases. In this study, we determined the CSF miRNA signature from A53T-transgenic mice and PD patients with same primers based on their highly conserved sequence. The miR-144-5p, -200a-3p and -542-3p were found to be significantly up-regulated in both A53T-transgenic mice and the CSF samples of PD patients. Similarly, it has been reported that miR-144-5p changed in the brain of Huntington disease and blood of AD [76, 77].The miR-200a-3p was involved in the regulation of neuronal differentiation and proliferation and miR-542-3p up-regulated in blood after ischemic stroke, intracerebral hemorrhage, and kainate seizures [78, 79]. In our results, miR-144-5p, -200a-3p and -542-3p were further selected by their differential expression of high-abundance in sequencing. The ROC analysis confirmed the sensitivity and specificity of these CSF markers are enough to distinguish the PD from healthy control. The ordinal regression analysis found their expression significant increased across pathologic severity of PD. And only the raised tendency of miR-542-3p was influenced by different smoking habit. These results supported these miRNA as independent factors may be the ideal biomarker-assisted diagnosis of PD. However, this topic still requires further investigation. In our study, we screened the similar miRNAs from PD patients and animal model to improve the repeatability, and some miRNAs were also reported in other study enrolled the patient from difference ethnicity, geography and had different inclusion and exclusion criteria.

In conclusion, this study described the miRNA signature in the midbrain of α-synuclein mutant mice by deep sequencing and predicted their functional network. Our investigation selected the potential miRNAs in CSF that were associated with PD. Of these miRNAs, the down-regulation of miR-196a-5p, -196b-5p, -10b-3p, -10a-5p, -615-3p, and -505-5p and the up-regulation of miR-144-5p, -542-3p, -200a-3p, -182-5p and -451a were further confirmed in the CSF of PD patients. The ROC results and ordinal regression analysis further suggested the miR-200a-3p, miR-144-5p and miR-542-3p may be potential biomarkers for PD prediction.

## MATERIALS AND METHODS

### Animal and clinical studies

All mice were used in accordance with the Animal Ethics Guidelines of the Institutional Animal Care Committee of Sun Yat-sen University (No. 20120112178). The transgenic mice B6; C3-Tg (Prnp-SNCA*A53T) 83Vle/J expressing A53T human α-synuclein, were originally obtained in Jackson Laboratory (JAX004479, USA). The breeding pairs were kindly offered by Dr. Ben Wu in the state key laboratory of medical genetics of Central South University. The hemizygous A53T mice on a mixed C57BL/6J × B6 background provided the transgenic and non-transgenic litter mates for study. DNA was purified from the tail by a DNA extraction kit (Beyotime, Beijing, China) with genotype identification (5′-TGTAGGCTCCAAAACCAAGG-3′, 5′-TGTCAGGATCC ACAGGCATA-3′). PD patient tissues were collected from the PD center of the First Affiliated Hospital of Sun Yat-Sen University and the Department of Neurology of Guangdong Brain Hospital between 2014 and 2015. All patients were diagnosed according to the Criteria of United Kingdom's Parkinson's disease Society [49]. The CSF samples of forty-four PD patients and forty-two healthy controls were collected from South China. The patient information was showed in Table 1. Inclusion criteria were: disease duration less than 3 years, early PD stage [1 ≥ H&Y ≥ 2.^5^], MMSE (Mini Mental State Examination) score > 26 corrected for age and education. Exclusion criteria were: other neurological disorders or psychiatric disease, familial neurodegenerative disease, concomitant systemic diseases as diabetes and other cardio-cerebrovascular diseases. All subjects were identified from the Han ethnic population in Guangdong province, China, approved by the medical ethics committee of Sun Yet-set University (No. 20120603014).

### Open field test and western blot and immunostaining

A truScan™ activity monitor (Coulbourn Instruments, Whitehall, PA, USA) was used to record mouse movement in a transparent Plexiglas box (30 × 30 × 30 cm) for 20 min each. Once mouse randomly was placed in the box, the travel distance was recorded in the field (4 cm from the middle line) [80]. Male mice were tested 3 times individually for wild-type and A53T transgenic mice (*n* = 6). The animal midbrain was homogenized in RIPA buffer (Cell Signaling, Beverly, MA, USA) containing protease inhibitor (Roche, Basel, Switzerland) and phosphatase inhibitor (PhosSTOP; Roche), and the protein concentrations were measured by a BCA protein assay (Thermo Fisher Scientific Inc., IL, USA). Western blot analysis was conducted with mouse anti-α-synuclein (1:500, Millipore, MABN826, USA), phosphorylated anti-α-synuclein (1:500, Millipore, AB5038, USA), β-actin antibody (1:1,000, Abcam, ab8227, Cambridge, UK), and horseradish peroxidase (HRP)-labeled secondary antibodies (KPL, MD, USA) in WT and A53T groups. Proteins were detected by the SuperSignal® West Pico Chemiluminescent Substrate (Thermo Fisher Scientific Inc., IL, USA) and quantified by Scanner EPSON Perfection V700 Photo.

Mice brains were perfused with 4% paraformaldehyde and dehydrated by a sequential sucrose gradient from 10% to 30%. The brain coronal section was blocked with 10% goat serum in 0.01 M phosphate-buffered saline (PBS) containing 0.1% Triton X-100 for 1 h. Slices were incubated with human α-synuclein (1:1000, Millipore, MABN826, USA) antibody and rabbit tyrosine hydroxylase (TH) antibody (1:400, Millipore, AB6211, USA) overnight at 4°C, washed three times with 0.01 M PBS, and then incubated with Alexa Fluor 488 (green) and 594 (red) conjugated secondary antibodies. The positive staining were visualized by the EVOS® FLoid® Cell Imaging Station (Thermo Scientific, USA). According to the study before [81], the TH positive cells in SN were counted in 15 μm coronary midbrain sections. Every sixth section from AP: -2.70 to AP: - 3.88 mm and a total number of 11 sections was collected. The TH positive cells on each side were counted and combined under the blinded genotype to the counter used the Stereo Investigator software (MBF Bioscience, Williston, VT, USA) [48].

### Deep sequencing

The 12-month-old mice were decapitated after deeply anesthetized with isoflurane (Baxter Healthcare, Deerfield, IL, USA). Total RNA was purified from midbrain tissues of one-year-old mice using TRIzol reagent (Invitrogen), concentrated by Qiagen RNeasy MinElute Cleanup Kit (Qiagen, Valencia, CA, USA), quantified (Nanodrop; Thermo Fisher, Uppsala, Sweden) and evaluated by RNA electrophoresis. RNA absorbance was measured between the range of 1.90 to 2.02 using an Agilent 2100 Bioanalyzer, with an RNA integrity number (RIN) ≥ 2.6 and RNA concentrations ≥ 3.0 mg/l. Small RNA libraries were constructed following the instruction of TruSeq Small RNA Sample Preparation Kits (Illumina, Inc., Hayward, CA, USA) and purified for deep sequencing with single-end reads of 36 bases on the Illumina HiSeq 2500 (Huada, Shenzhen, China, Supplementary Figure 1) [82, 83]. The annotated and unannotated reads from RNA sequencing were detected by Bowtie (v0.12.7) and the miRDeep2 software to analyze the mapped reads and predict novel miRNAs. The miRNAs from miRDeep2 were further analyzed by the Bioconductor DESeq v. 2.0 package using a *p* value < 0.05 and fold change (FC) > 2 [84]. The prediction analysis of miRNA-targeted genes was based on COG (Cluster of Orthologous Groups of proteins), GO (Gene Ontology), Swiss-Prot (a manually annotated and reviewed protein sequence database), Nr (NCBI non-redundant nucleotide sequences) and KEGG (Kyoto Encyclopedia of Genes and Genomes) databases.

### Quantitative real-time PCR

Quantitative real-time PCR (qRT-PCR) was performed following the MIQE guidelines [85]. Total RNA, purified from midbrain tissues of 12-month-old mice and CSF of human, was extracted by a mirVana PARIS Kit (Ambion, PN AM1556) and converted to cDNA using a TaqMan® MicroRNA Reverse Transcription Kit (ABI, USA). A high-capacity cDNA reverse transcription kit (ABI, USA) was used for random primer scheme of miR-615-5p and miR-196a with low concentration [86, 87]. Selected miRNAs were quantified on an ABI Prism 7500 system (Applied Biosystems, Warrington, UK). The stem-loop RT and PCR primers were ordered from TIANGEN Biotech (Supplementary Table 1). U6 in midbrain and miR-34 in CSF were used as an internal control following the 2^−ΔΔCT^ and ΔCT methods, respectively [33, 88].

### Statistical analysis

Statistical analyses were performed using SPSS 13.0 (SPSS Inc., Chicago, IL) and Prism 5.0 (GraphPad Software Inc, La Jolla, CA). The normally distributed data were compared by Student's t test, whereas the non-Gaussian data were analyzed using Wilcoxon-Mann-Whitney tests. In the deep sequencing analysis, we calculated the normalized TPM as actual miRNA count/total count of clean tags×1,000,000 [89]. The FC value was calculated following FC = log_2_ (TPM of A53T/TPM of control). The *p*-value for the analysis of miRNAs was adjusted using the Benjamini-Hochberg approach. The enrichment factor (EF), defined as the ratio of observed to expected for a given enrichment class, was used for gene-enrichment analysis, and *p*-values < 0.05, as determined using Fisher's exact test, was used to assess statistical significance. The ROC analysis and area under ROC curve (AUC) were performed to assess the possibility of miRNA concentration as biomarkers for PD diagnosis. Youden index was used for the selection of cut-off point. According to the study before, the ordinal logistic regression was used for the analysis between miRNAs expression and H&Y scales by STATA (version 13, StataCorp, College Station, Texas) [31]. The p values were corrected by the Bonferroni method in multiple testing. All the *p* values were 2-tailed.

## SUPPLEMENTARY MATERIAL


