# Supplementary material for: MicroRNA expressing profiles in A53T mutant alpha-synuclein transgenic mice and Parkinsonian
Source: Oncotarget. 2016 Dec 11;8(1):15–28. doi: 10.18632/oncotarget.13905 (PMC5352072; doi:10.18632/oncotarget.13905)
Supplement: Supplementary file 1 [file oncotarget-08-15-s001.pdf]

MicroRNA expressing profiles in A53T mutant alpha-synuclein transgenic mice and parkinsonian

Supplementary Material

Supplementary Table 1. The miRNA primers used for qRT-PCR

miRNA name		Primer sequence
miR-196a-5p	Forward	5′-CGTCAGAAGGAATGTGCACAG -3′
	Reverse	5′-ACCTGCGTAGGTAGTTTCATGT -3′
miR-196b-5p	Forward	5′-TAGGTACCACTTTATCCCGTTCACCA -3′
	Reverse	5′-ATCTCGAGGCAGGGAGAGAGGAAT AA -3′
miR-10b-3p	Forward	5′-TCGGCGTACCCTGTAGAACCGAAT -3′
	Reverse	5′-GTCGTATCCAGTGCAGGGTCCGAGGT-3′
miR-10a-5p	Forward	5′-ACGGGTACCCTGTAGATCCG-3′
	Reverse	5′-CAGTGCAGGGTCCGAGGTAT-3′
miR-615-3P	Forward	5′-TAYGGGGTAGATATGGTTGTTAATTTT-3′
	Reverse	5′-CTATACATCCCRAAATTCAAAAAAACC-3′
miR-200a-3p	Forward	5-CCGCCGTAATACTGTCTGGTG-3′
	Reverse	5′-CGCAGGGTCCGAGGTATTC -3′
miR-505-5P	Forward	5′- GGGAGCCAGGAAGTATTGATGT-3′
	Reverse	5′-UUCUCCGAACGUGUCCGUTT-3′
miR-182-5p	Forward	5′-TGCGGTTTGGCAATGGTAGAAC-3′
	Reverse	5′-CCAGTGCAGGGTCCGAGGT-3′
miR-451a	Forward	5′-AAACCGTTACCATTACTGAGTT-3′
	Reverse	5′-CGCTACGTAACGGCATGACAGTG-3’
miR-144-5p	Forward	5′-GCTGGGATATCATCATATACTG-3′
	Reverse	5′-CGGACTAGTACATCATCTATACTG-3’
miR-542-3p	Forward	5′-ATCGTACGTGGGTGTGACAGA-3′
	Reverse	5′-GCAGGGTCCGAGGTATTC-3’
miR-24	Forward	5’-ACACTCCAGCTGGGTGGCTCAGTTCAGCAG-3’
	Reverse	5’- CTCAACTGGTGTCGTGGAGTCGGCAATTCAG-3
U6	Forward	5′-CTCGCTTCGGCAGCACA-3′
	Reverse	5′-AACGCTTCACGAATTTGCGT-3′

Supplementary Table. 2. Small RNA distribution in sequenced RNA libraries.

The sequence alignments in A53T and WT mice were compared using Silva, GtRNA db, Rfam and Repbase databases though the Bowtie software.

Types	WT		A53T	
	Number	Percentage	Number	Percentage
Total	9,476,822	100%	9,465,943	100%
rRNA	359,171	3.79%	356,533	3.77%
scRNA	0	0.00%	0	0.00%
snRNA	1,516	0.16%	14,085	0.15%
snoRNA	117,512	1.24%	116,799	1.23%
tRNA	90,977	0.96%	89,968	0.95%
Repbase	171,530	1.81%	169,366	1.79%
Unannotated	8,736116	32.14%	8,719,192	32.16%

Supplementary Table.3. Top-10 pathways in KEGG

The predicted candidate genes were mapped back to the Kyoto Encyclopedia of Genes and Genomes (KEGG) pathways, the number on each pathway was calculated and the top-10 ranking KEGG pathways are shown. Data includes the KEGG pathway name, pathway identifier (KO ID), fraction of interacting genes that are annotated to the pathway (cluster frequency), fraction of genes in the genome annotated to the pathway (genome frequency) and the corrected p-value of hypergeometric distribution (Benjamini & Yekutieli method).

KEGG pathway	KO ID	Cluster frequency	Genome frequency	P value
Neuroactive ligand receptor interaction	ko04080	306/5985	315/6469	0.000301058
Focal adhesion	ko04510	191/5985	195/6469	0.000747581
Gap junction	ko04540	87/5985	87/6469	0.001099635
Protein digestion and absorption	ko04974	76/5985	76/6469	0.002616091
Osteoclast differentiation	ko04380	112/5985	114/6469	0.007036206
ErbB signaling pathway	ko04012	86/5985	87/6469	0.008949007
Acute myeloid leukemia	ko05221	57/5985	57/6469	0.011647199
Inositol phosphate metabolism	ko00562	56/5985	56/6469	0.012597991
Axon guidance	ko04360	121/5985	124/6469	0.013931966
Drug metabolism- other enzymes	ko00983	54/5985	54/6469	0.014738195

Supplementary Table.4. The qRT-PCR miRNA sequence

The sequencing of miRNAs from human and mouse were provided by the NCBI database (http://www.mirbase.org).

miRNA name	Species	Sequence shown in miRbase database
miR-196a-5p	Human	UAGGUAGUUUCAUGUUGUUGGG
	Mouse	UAGGUAGUUUCAUGUUGUUGGG
miR-196b-5p	Human	UAGGUAGUUUCCUGUUGUUGGG
	Mouse	UAGGUAGUUUCCUGUUGUUGGG
miR-10a-5p	Human	UACCCUGUAGAUCCGAAUUUGUG
	Mouse	UACCCUGUAGAUCCGAAUUUGUG
miR-10b-3p	Human	ACAGAUUCGAUUCUAGGGGAAU
	Mouse	CAGAUUCGAUUCUAGGGGAAUA
miR-615-3P	Human	UCCGAGCCUGGGUCUCCCUCUU
	Mouse	UCCGAGCCUGGGUCUCCCUCUU
miR-200a-3p	Human	UAACACUGUCUGGUAACGAUGU
	Mouse	UAACACUGUCUGGUAACGAUGU
miR-429-3p	Human	UAAUACUGUCUGGUAAUGCCGU
	Mouse	UAAUACUGUCUGGUAAUGCCGU
miR-182-5p	Human	UUUGGCAAUGGUAGAACUCACACU
	Mouse	UUUGGCAAUGGUAGAACUCACACCG
miR-451a	Human	AAACCGUUACCAUUACUGAGUU
	Mouse	AAACCGUUACCAUUACUGAGUU
miR-505-5P	Human	GGGAGCCAGGAAGUAUUGAUGU
	Mouse	GGGAGCCAGGAAGUAUUGAUGUU
miR-144-5p	Human	GGAUAUCAUCAUAUACUGUAAG
	Mouse	GGAUAUCAUCAUAUACUGUAAGU
miR-542-3p	Human	UGUGACAGAUUGAUAACUGAAA
	Mouse	UGUGACAGAUUGAUAACUGAAA

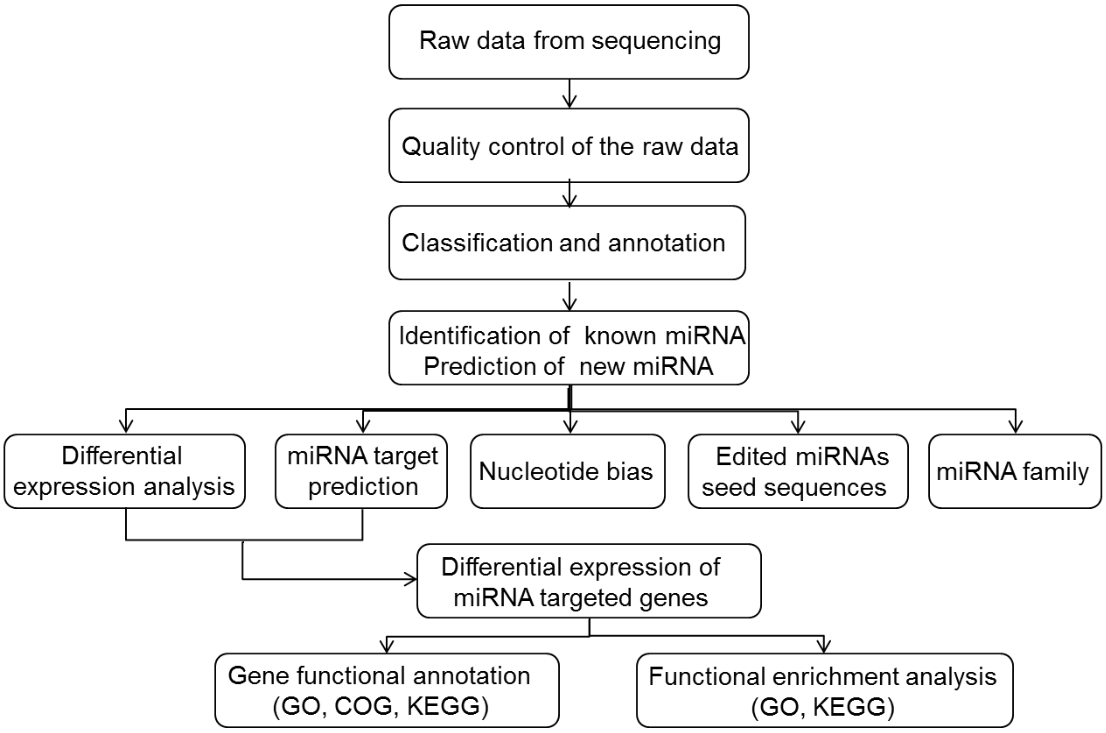

Supplementary Fig. 1. The procedure for deep sequencing

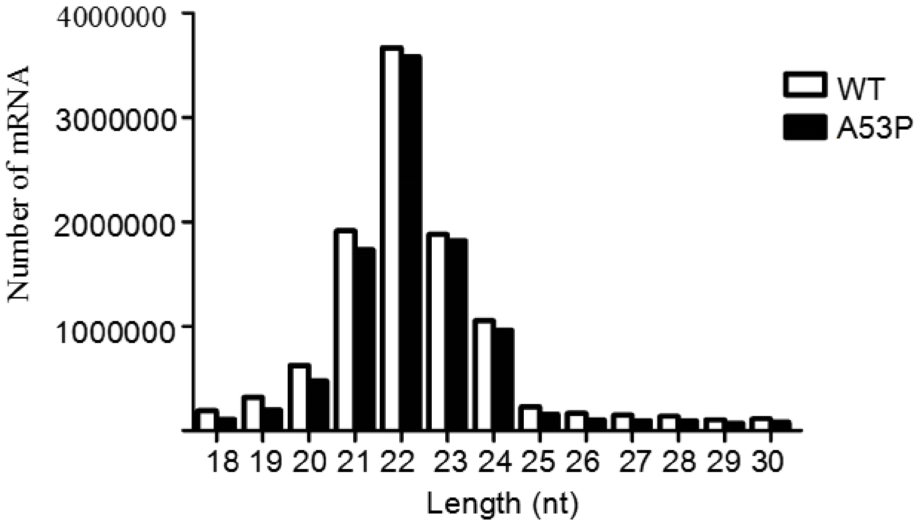

Supplementary Fig.2. Distribution of small RNAs in wildtype and A53T-transgenic mice.

The distribution of small RNAs arranged from 18 to 30 nt. The read lengths are represented on the vertical and the total read counts of RNA showed on the horizontal axis.

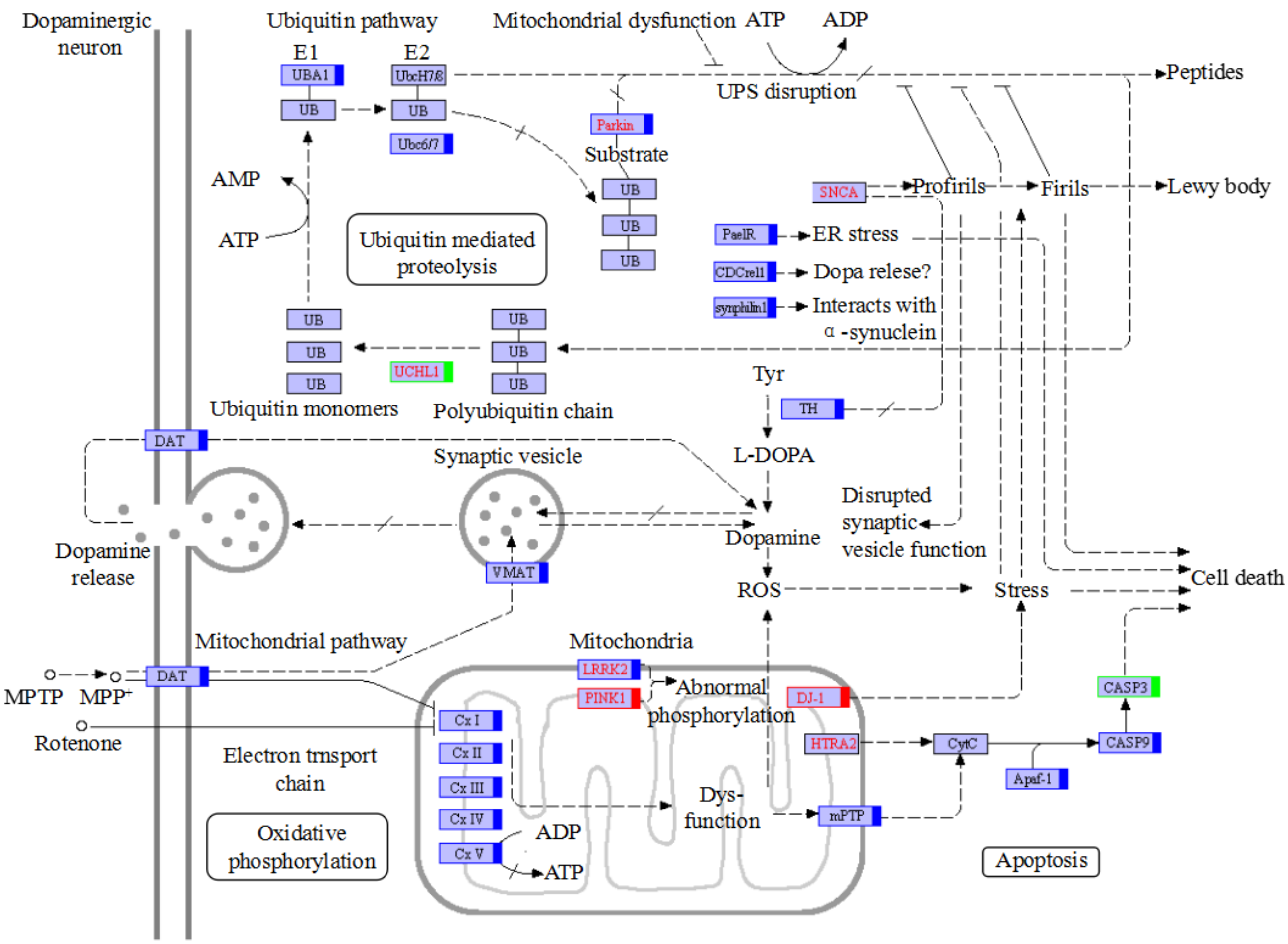

Supplementary Fig.3. Annotated pathways of KEGG in Parkinson’s disease

Pathways of regulated genes indicated enrichment within the interacting genes for the pathways in Parkinson’s disease. The relevant genes were mapped on the Kyoto Encyclopedia of Genes and Genomes (KEGG) pathways and are indicated with different colors. Green: related with the down-regulated targeted genes. Red: related with the up-regulated targeted genes. Blue: related with both up-regulated and down-regulated targeted genes.

Variables	miR-200a-3p			miR-144-5p			miR-542-3p		
	Coefficient	95% CI	P	Coefficient	95% CI	P	Coefficient	95% CI	P
Age (>55, ≤55)	1.84	-1.96-5.65	0.34	-0.37	-0.93-0.19	0.19	-0.27	-1.44-0.90	0.65
Gender (Male, Female)	0.68	-2.66-4.01	0.69	0.22	-0.36-0.79	0.47	0.36	-0.83-1.54	0.56
Smoking (Current, Ever, Never)	3.62	0.28-6.96	0.03	-0.06	-0.57-0.46	0.83	-0.26	-1.396-0.87	0.65

Supplementary Fig.4. Correlation of CSF miRNAs Levels with H&Y scale by ordinal regression analysis
